# Antiplasmodial, antioxidant and immunomodulatory activities of ethanol extract of *Vernonia amygdalina* del. Leaf in Swiss mice

**Published:** 2016

**Authors:** Ehimwenma Sheena Omoregie, Anirban Pal

**Affiliations:** 1 *Department of Biochemistry, Faculty of Life Sciences, University of Benin, PMB 1154, Benin City, Nigeria *; 2 *In vivo Animal Testing Facility, Central Institute of Medicinal and Aromatic Plants (CIMAP), Lucknow, India*

**Keywords:** *Antiplasmodial*, *Antioxidant*, *Immunomodulatory*, *Vernonia amygdalina*, *HPTLC*

## Abstract

**Objective:**

*Vernonia amygdalina* (*V. amygdalina*) leaf is locally employed in the Southern region of Nigeria in the treatment of malaria infection. This study evaluated the *in vivo* antiplasmodial, antioxidant and immunomodulatory effect of ethanol extract of *V. amygdalina* leaf.

**Materials and Methods:**

The active principles of the dried leaf were extracted with ethanol. For quality validation, chemical finger-print of the extract was performed through high performance thin layer chromatography (HPTLC). The extract was assessed for antiplasmodial activity by the standard four-day suppressive test on *Plasmodium berghei* (ANKA) infected male Swiss mice (six weeks old) placed into five groups of six animals each.

**Result:**

The absorption spectra from the HPTLC revealed several peaks suggesting presence of some bioactive compounds. Results from the *in vivo* study showed that the ethanol extract of the plant leaf was significantly active against *P. berghei* in a dose-dependent manner with the minimum and maximum activity observed in the mice treated orally with 100mg/kg (% inhibition of 23.7%) and 1000 mg/kg (% inhibition of 82.3 %) of the extract, respectively, on day four of the study. There was also a dose-dependent decrease (p<0.05) in some oxidative stress indices including nitric oxide and lipid peroxidation levels in the extract treated groups as against the non-treated infected group which had high levels of these parameters. The pro-inflammatory cytokines (TNF-α and IFN-ɣ) levels were also considerably low in the extract treated groups relative to the non-treated infected group.

**Conclusion:**

The results suggest that ethanol extract of *V. amygdalina* leaf was active, with some immunomodulatory effect, against *P. berghei* infection.

## Introduction

Malaria is one of the most pathogenic diseases in endemic areas of Africa, Asia and Latin America with more than (350-500) million people in Africa infected by malaria parasite, commonly *Plasmodium falciparum* (one of the five species of malaria parasites infecting humans) with 80 million reported clinical cases and more than 2 million deaths annually (WHO, 2005 ▶; Adegbolagun et al., 2014 ▶). The problem is further compounded by the upsurge in the resistance strain of the parasite. Thus, the continuous search for novel and more effective antimalarial compounds especially from medicinal plants extracts is of utmost importance in view of the success of artemisinin, the active principle of an ancient Chinese herbal remedy for fevers (Trager and Jensen, 1997 ▶; Chung et al., 2009 ▶; Osamor, 2010 ▶). 

The traditional method of malaria treatment is a promising source of new antimalarial compounds. In Africa, the use of indigenous plants plays an important role in the treatment of malaria by providing good sources for the detection of novel antiplasmodial compounds. The therapeutic properties ascribed to most medicinal plants have been linked to their phytochemical constituents (Wright, 2005 ▶). 

*Vernonia amygdalina* (*V. amygdalina*), commonly called *bitterleaf*, *ewuro, *is an edible rainforest plant and is one of the locally used plants in the treatment of malaria infection (Erasto et al., 2007 ▶). Members of the genus are good sources of sesquiterpenes lactones which have been reported to possess numerous activities such as insect antifeedant, antifungal, cytotoxic, antitumoral and antiplasmodial (Kumari et al., 2003 ▶). The occurrences of steroidal saponins, tannins, alkaloids and flavonoids have also been reported (Akinpelu 1999 ▶; Erasto et al., 2006 ▶). Based on the myriad ethnomedicinal uses of *V. amygdalina*, the present study, therefore, evaluates the antiplasmodial, antioxidant and immunomodulatory properties of ethanol extract of *V. amygdalina* leaf in Swiss mice.

## Materials and Methods

**Plant material**

*V. amygdalina* leaves were collected during the rainy season (May–June, 2013), from a private farm located at Ugbowo Quarters, Benin City, Nigeria. The fresh leaves were authenticated by a Botanist, at the Department of Plant Biology and Biotechnology, University of Benin, Benin City, Nigeria. A voucher specimen (TK/37682) was deposited in the herbarium of the same department. 

**Preparation of ethanol extract**

*V. amygdalina* leaves were washed, air-dried, macerated and then extracted with ethanol at room temperature for 72 hours with stirring at intervals. The extracts obtained were concentrated to dryness at 40^o^C using a rotary evaporator under reduced pressure (Ayoola et al., 2008 ▶). The dried extracts were weighed and then stored at 4^o^C for subsequent analyses. 

**HP-TLC chemical finger-printing of plant extract**

100mg of the plant extract was dissolved in 1.0ml of methanol by means of ultra-sonication for 30 minutes, and centrifuged at 8000rpm for 10 minutes. The supernatant obtained was used for the HPTLC chemical profile with chloroform: methanol (9:1, v/v) serving as the mobile phase. Sample application plates were developed for one hour in a Camag 10 × 10 twin trough glass solvent developing chamber initially pre-saturated with the mobile phase. The plates were air-dried and scanned using a Camag TLC Scanner model 3 (with slit size 10 × 0.40mm and wavelength of 254nm) equipped with Wincats software and absorption-reflection scan mode. The calibration curve of peak area vs. concentration was thereafter prepared.

***In vivo*** **antiplasmodial activity assay**

The *in vivo* antimalarial activity of the extracts was determined by the classic four – day suppressive test against *P. berghei*, ANKA strain, in mice (Peters, 1992 ▶). Briefly, thirty six Swiss female mice (20–22) g, in groups of six animals, were inoculated with 1 × 10^6^ infected red blood cells intraperitoneally on day 0. After 2 hours of inoculation, the mice in groups III, IV and V were treated orally with the extracts suspended in carboxyl methyl cellulose (CMC) at different concentrations (100 mg/kg, 300 mg/kg and 1000 mg/kg body weight, respectively). While group II infected mice were treated orally with chloroquine (10 mg/kg body weight). Group I infected animals were left untreated throughout the duration of the study. 

The treatment was repeated for 3 consecutive days from day 0. Blood films were taken on the fourth day (96 hours after the first dose), Giemsa stained and examined microscopically and the level of parasitaemia was determined by counting 2000 erythrocytes. The extract activity was determined by percent reduction of parasitaemia in the treated groups compared with the untreated infected mice. The ED_50_ representing 50% suppression of parasites when compared with untreated control was estimated by a non-linear dose-response curve fitting analysis (Graph Pad Prism version 6 statistical software). Crude extracts with ED_50_ values > 50 mg/kg were considered to be inactive. Death occurring before day 5 of infected and treated mice was regarded as toxic death. 

All of the experimental protocol was in accordance with internationally accepted guidelines for animals use and care (EEC Directive of 1986; 86/09/EEC; National Institutes of Health Publication 85-23, revised 1985). The experiments and procedures employed in this study were also reviewed and approved by the Animal Care Ethics Committee of the University of Benin, Benin City, Nigeria.

**Collection of blood and organs**

 Animals were fasted overnight and sacrificed by cervical dislocation. Blood was collected from the heart via a syringe and the serum obtained from the whole blood was stored at -80^0^C. The tissues (liver, brain, spleen and kidney) were removed at once, blotted dry, weighed and stored as before.

**Preparation of tissue homogenate**

1g of the tissue was homogenized in 10ml of ice-cold physiological saline (0.9 %) to obtain 10% (w/v) homogenates. The resulting homogenate was centrifuged at 5,000g for 10 minutes and the supernatant obtained was used for subsequent assays.

**Assay of lipid peroxidation**

Lipid peroxidation in the tissue homogenate was determined indirectly by estimating thiobarbituric acid reactive substances (TBARS) levels, which are indicators of membrane lipid peroxidation. The values for TBARS were reported as malondialdehyde (MDA) and quantified using a Molar extinction coefficient of 1.5 × 10^5^ M cm-1, expressed as mmole MDA g-1 of tissue (Gutteridge and Wilkins, 1982 ▶).

**Estimation of nitric oxide**

The nitric oxide (NO) level was determined by the Griess method (Green et al., 1982 ▶) as reported by Awasthi et al., (2003). 100ul of serum / brain homogenate were mixed with 100ul Griess reagent which consists of equal volumes of 1% sulfanilamide and 0.1% naphthylethyl-enediamine dichloride in 2.5% H_3_PO_4_ in a 96 microliter plate. Absorbance at 540nm was measured using an ELISA plate reader (Molecular Devices Emax Miroplate Reader, Downington, PA) and the concentration of NO was calculated from a standard curve. 

**Estimation of tumour necrosis factor–alpha (TNF-) and interferon–gamma (IFN-)**

Tumour Necrosis Factor – alpha (TNF-) and Interferon–gamma (IFN-) were estimated by the use of Thermo Scientific ELISA kit for quantification of mouse TNF- and IFN- in serum. Absorbance was measured on an ELISA plate reader set at 450 and 550nm. 

**Statistical analysis**

The data were analyzed using Graph Pad Prism version 4 software and the results were expressed as means ± standard error of mean (SEM). One-way analysis of the variance (ANOVA) was performed to check for differences between the groups mean. The significant differences between the means were compared non-parametrically by the Tukey’s test. P values < 0.05 were regarded as significant.

## Results

**Figure 1:**
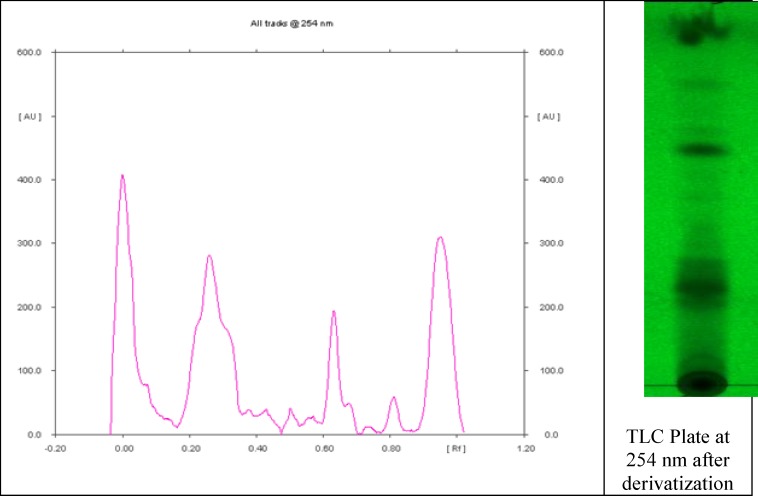
Chemical fingerprint of ethanol extract of *Vernonia amygdalina* leaf

Figure 1 represents the HP-TLC profile of ethanol extract of *V. amygdalina*. The HP-TLC profile revealed several peaks suggesting the presence of some bioactive compounds. Clear peaks were observed with different Rf values ranging from -0.00 to 0.95 and percentage (%) yield of between 0.054 % to 31.88 %. 

**Figure 2:**
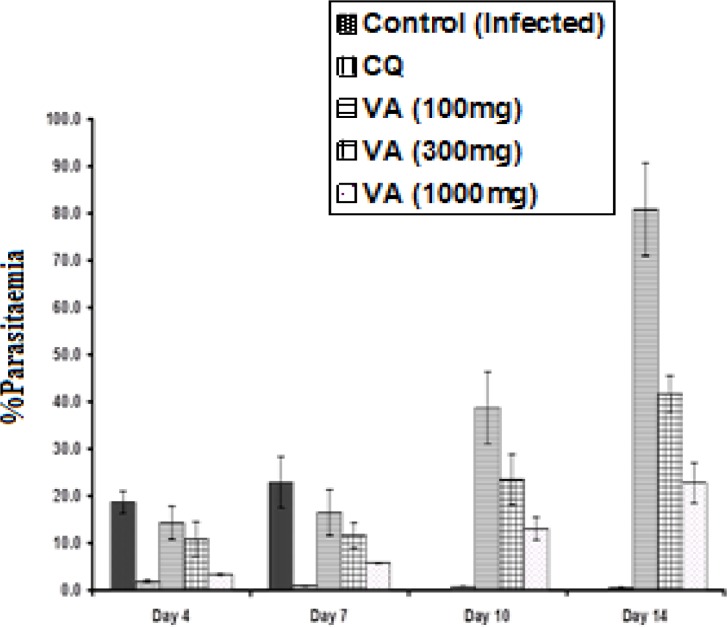
Effects of ethanol extract of *V. amygdalin*a leaf and chloroquine on* in vivo* growth of *P. berghei* in mice. Data are mean count ± S.D. (n = 6 for each group). CQ = chloroquine treated group; Control (infected) = non-treated infected group; VA (100mg) = *V. amygdalina* extract (100mg/kg body weight) treated group; VA (300mg) = *V. amygdalina* extract (300mg/kg body weight) treated group; VA (1000mg) = *V. amygdalina* extract (1000mg/kg body weight) treated group

Figure 2 shows the percentage parasitaemia level of the infected mice treated with the extract and chloroquine (CQ). The percentage (%) parasitaemia was significantly high (p<0.05) in the non-treated infected group (18.6 ± 0.16%) but low for the CQ treated mice (1.8 ± 0.03%) from day 0 to 4 of the study. On the other hand, the treatment of the infected mice with various doses (100, 300 and 1000 mg/kg body weight) of the ethanol extract of *V. amygdalina *leaf resulted in a dose-dependent reduction in parasitaemia level (14.2 ± 0.23%, 10.0 ± 0.47%, and 3.3 ± 0.08%, respectively) from day 0 to 4 and then it increased from day 7 to 14 of the study. The plant extract, thus, showed moderate antimalarial activity when compared with CQ with their respective ED_50_ of 242.5 ± 4.8 mg/kg and 10.67 ± mg/kg. The mean survival times of the mice administered 100, 300, and 1000 

mg/kg/day of *V. amygdalina* extract, 20mg/kg/day chloroquine, and saline (infected control) were 14.0, 18.0, 20.0, 28.0, and 8.0 days, respectively. The CQ treated mice therefore, recorded no death from day 0 to day 28 of the experiment. 

**Figure 3:**
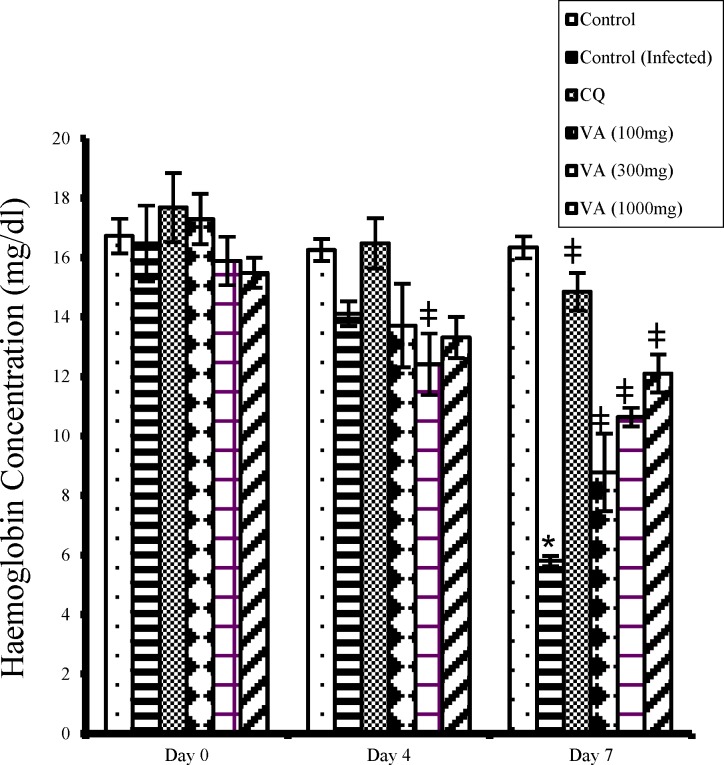
Effect of ethanol extract of *V. amygdalina* leaves and chloroquine on hemoglobin level in *P. berghei* infected mice. Data represent mean ± SEM (n = 6 for each group). CQ = chloroquine treated group; Control = uninfected group treated with physiological saline; Control (infected) = non-treated infected mice treated with the vehicle (carboxylmethyl cellulose); VA (100mg) = *V. amygdalina* extract (100mg/kg body weight) treated group; VA (300mg) = *V. amygdalina* extract (300mg/kg body weight) treated group; VA (1000mg) = *V. amygdalina* extract (1000mg/kg body weight) treated group. * = p < 0.05) when compared with control; ǂ = p < 0.05 when compared with non-treated infected group (control (infected))

Hemoglobin concentration decreased significantly (p<0.05) in the non-treated infected mice (negative control) from day 0 to day 7 of the study (Figure 3). The extract treated groups, however, showed significant increase (p<0.05) in hemoglobin levels when compared with the negative control, but remained less than that of the normal non-infected mice (positive control). The CQ treated mice also had normal hemoglobin concentration that was comparable to that of the positive control (uninfected mice). 

**Figure 4:**
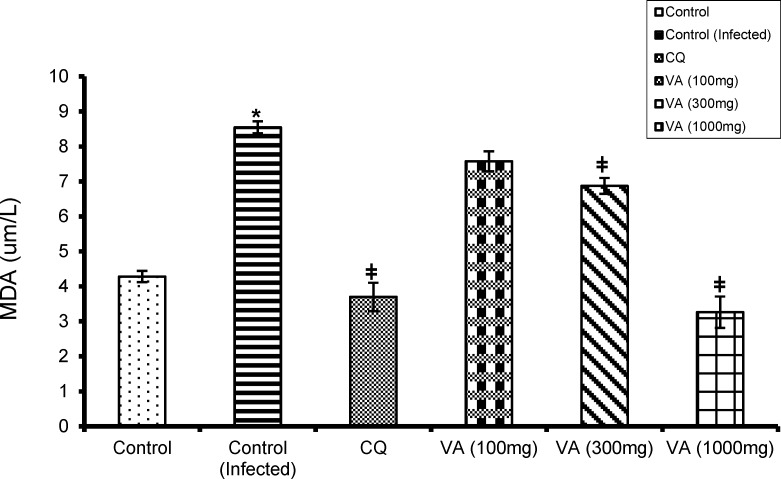
Effect of ethanol extract of *V. amygdalina* leaves and chloroquine on lipid peroxidation level in *P. berghei* infected mice. Data represent mean ± SEM (n = 6 for each group). CQ = chloroquine treated group; Control (infected) = non-treated infected group; VA (100mg) = *V. amygdalina* extract (100mg/kg body weight) treated group; VA (300mg) = *V. amygdalina* extract (300mg/kg body weight) treated group; VA (1000mg) = *V. amygdalina* extract (1000mg/kg body eight) treated group. = non-treated infected mice treated with the vehicle (carboxylmethyl cellulose); VA (100mg) = *V. amygdalina* extract (100mg/kg body weight) treated group; VA (300mg) = *V. amygdalina* extract (300mg/kg body weight) treated group; VA (1000mg) = *V. amygdalina* extract (1000mg/kg body weight) treated group. * = p < 0.05 when compared with control; **ǂ** = p < 0.05 when compared with non-treated infected group (control (infected

Figure 4 shows the effect of CQ and extract on lipid peroxidation (LPO) level in normal and infected mice. There was a marked increase in LPO concentration in the untreated infected mice as against the normal control (p<0.05). Whereas, the CQ and extract treated groups showed significant reduction (p<0.05) in LPO level when compared with the normal control group. 

**Figure 5:**
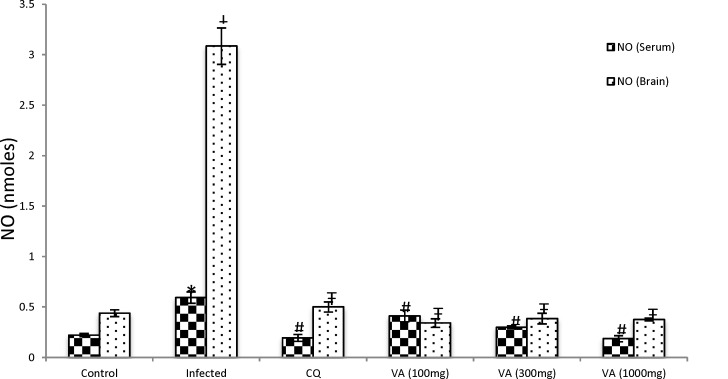
Effect of ethanol extract of *V. amygdalina* leaves and chloroquine on nitric oxide level in *P. berghei* infected mice. Data represent mean ± SEM (n = 6 for each group). CQ = chloroquine treated group; Control (infected) = non-treated infected group; VA (100mg) = *V. amygdalina* extract (100mg/kg body weight) treated group; VA (300mg) = *V. amygdalina* extract (300mg/kg body weight) treated group; VA (1000mg) = *V. amygdalina* extract (1000mg/kg body weight) treated group. = non-treated infected mice treated with the vehicle (carboxylmethyl cellulose); VA (100mg) = *V. amygdalina* extract (100mg/kg body weight) treated group; VA (300mg) = *V. amygdalina* extract (300mg/kg body weight) treated group; VA (1000mg) = *V. amygdalina* extract (1000mg/kg body weight) treated group. * = p<0.05 when compared with nitric oxide (NO) level of control (serum); ǂ = p < 0.05) when compared with NO level of control (brain); # = p<0.05 when compared with NO of non-treated infected group (infected - serum); Ŧ = p<0.05 when compared with NO of non-treated infected group (infected - brain)

The nitric oxide (NO) level was also significantly high (p < 0.05) in the serum and brain tissues of the non-treated infected infected mice when compared with the non-infected group (Figure 5). But treatment of the non-treated infected mice with different doses of the extract resulted in a dose-dependent decrease in NO level relative to the control (p<0.05). The CQ treated mice had normal NO level that was comparable with control levels. 

**Figure 6:**
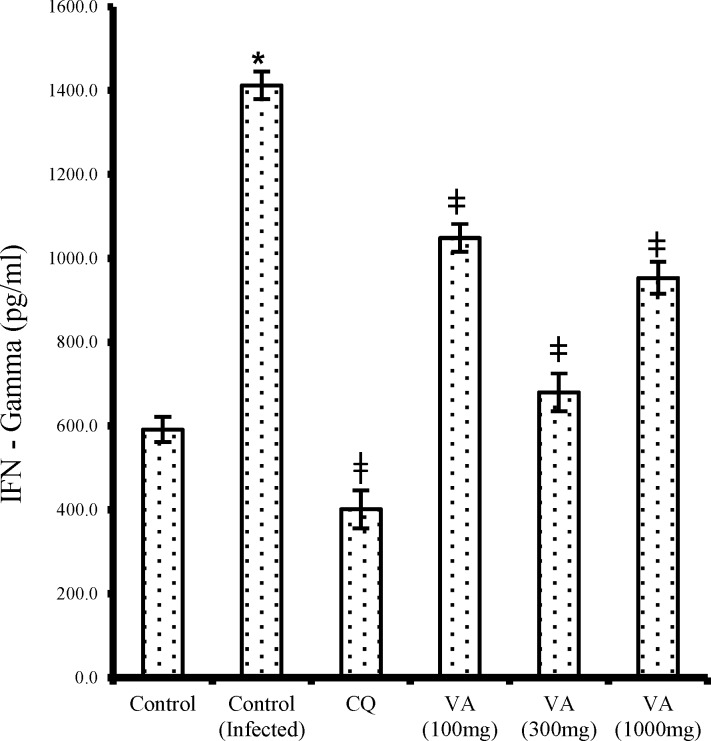
Effect of ethanol extract of *V. amygdalina* leaves and chloroquine on IFN-  level in *P. berghei* infected mice. Data represent mean ± SEM (n = 6 for each group). CQ = chloroquine treated group; Control (infected) = non-treated infected group; VA (100mg) = *V. amygdalina* extract (100mg/kg body weight) treated group; VA (300mg) = *V. amygdalina* extract (300mg/kg body weight) treated group; VA (1000mg) = *V. amygdalina* extract (1000mg/kg body weight) treated group. = non-treated infected mice treated with the vehicle (carboxylmethyl cellulose); VA (100mg) = *V. amygdalina* extract (100mg/kg body weight) treated group; VA (300mg) = *V. amygdalina* extract (300mg/kg body weight) treated group; VA (1000mg) = *V. amygdalina* extract (1000mg/kg body weight) treated group. * = p<0.05 when compared with control; **ǂ** = p<0.05 when compared with non-treated infected group (control (infected

**Figure 7:**
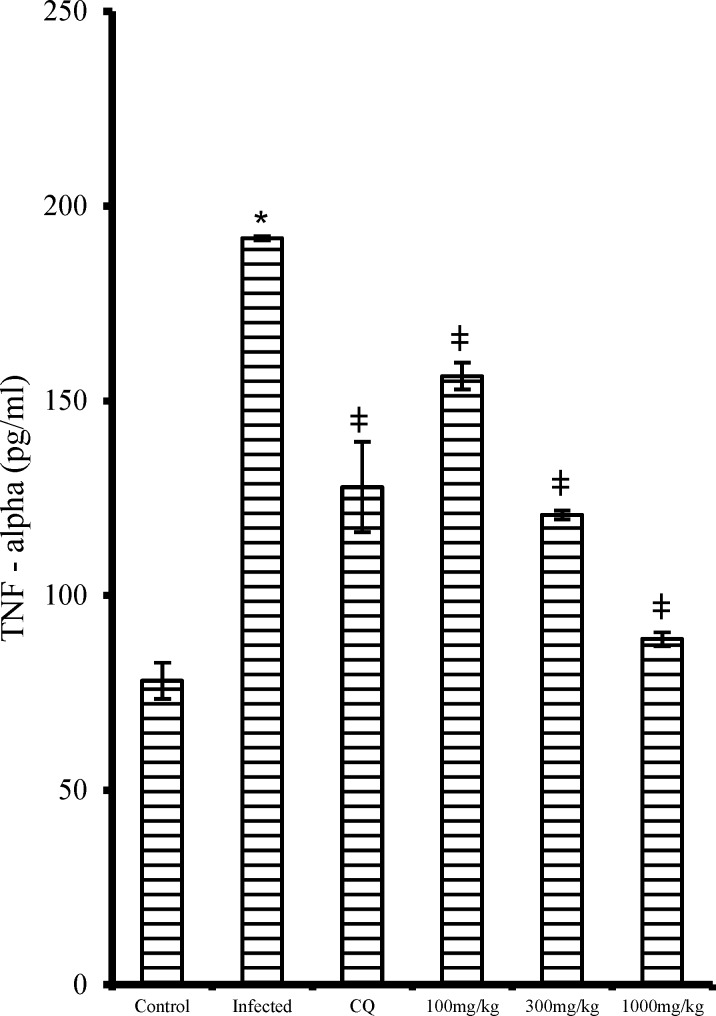
Effect of ethanol extract of *V. amygdalina* leaves and chloroquine on TNF-  level in *P. berghei* infected mice. Data represent mean ± SEM (n = 6 for each group). CQ = chloroquine treated group; Control (infected) = non-treated infected group; VA (100mg) = *V. amygdalina* extract (100mg/kg body weight) treated group; VA (300mg) = *V. amygdalina* extract (300mg/kg body weight) treated group; VA (1000mg) = *V. amygdalina* extract (1000mg/kg body weight) treated group. = non-treated infected mice treated with the vehicle (carboxylmethyl cellulose); VA (100mg) = *V. amygdalina* extract (100mg/kg body weight) treated group; VA (300mg) = *V. amygdalina* extract (300mg/kg body weight) treated group; VA (1000mg) = *V. amygdalina* extract (1000mg/kg body weight) treated group. * = p<0.05 when compared with normal control; **ǂ** = p<0.05 when compared with non-treated infected group (infected

The pro-inflammatory cytokines, serum interferon gamma (IFN-) and serum tumor necrosis factor – alpha (TNF-α) showed a dose-dependent decrease (p<0.05) in the extract treated mice in contrast to the non-treated infected mice (Figures 6 and 7, respectively). Likewise, the CQ treated group also had significantly reduced (p<0.05) IFN- and TNF-α levels when compared with that of the non-treated infected group.

## Discussion

This study assessed the antiplasmodial activity of ethanol extract of *V. amygdalina* (bitter leaf) leaf in *P. berghei* infected mice. The results revealed relatively reduced percentage (%) parasitaemia, on day 4, in the extract treated group as against the non-treated infected group which showed very high % parasitaemia. The antiplasmodial activity of the plant extract was found to be dose-dependent with the lowest antiplasmodial activity observed at 100 mg/kg and the highest activity at 1000 mg/kg body weight. The antiplasmodial activity of the extract was moderate when compared with that of the reference drug, CQ which showed complete suppression of parasitaemia to a non-detectable level. The complete parasite suppressive effect of CQ agrees with previous findings on *P. berghei* infected mice (Abdulelah and Zainal-Abidin, 2007 ▶; Park et al., 2008 ▶). 

The mean survival times of the mice administered various doses of the *V. amygdalina* extract, showed that the highest dose (1000mg/kg) corresponds with the highest survival time (20 days) which was again less than that of the reference drug CQ (28 days) with complete clearance of the parasite (aparasitaemia) observed in the latter. Nevertheless, the parasite suppressive ability of ethanol extract of *V. amygdalina* may be considered as good (with ED_50_ = 242.5 mg/kg) based on previous classification of antiplasmodial activity of crude extracts, which revealed that extracts with ED_50 _values of 500, 250, and 100mg/kg falls within moderate, good and very good activity, respectively (Syamsudin et al., 2007 ▶). The non-significant increase (p>0.05) in body weight of the infected mice treated with the extract at all dose levels suggests that it might have protected the mice from weight loss due to the presence of several nutrients and immune-modulatory substances, in addition to the anti-parasitic activity of the extract in the mice (Dikasso et al., 2006 ▶).

In malaria infection, TNF- is produced by monocytes / macrophages exposed to malaria parasites, the soluble antigens (such as glycosylphosphatidylinositol (GPI)) and malaria pigment (hemozoin). Other cytokines such as interleukins, IFN- as well as nitric oxide intermediates (NOI) and reactive oxygen intermediates (ROI) are involved with TNF- in the control of malaria infection. TNF- is one of the effector molecules with pleiotropic effects released by natural cytotoxic cells as well as from antigen–specific cytotoxic T lymphocytes (CTL) and helper T cells in the control of tumours and viral, bacterial, parasite and fungal infections (Richards, 1997 ▶; Depinay et al., 2011 ▶). A TNF super family, consisting of 19 members signaling through 29 receptors, induces other cytokines and enzymes that add to the inflammatory cascade (Clark et al., 2006 ▶). 

TNF- has been said to play both beneficial and detrimental roles during malaria infection (Depinay et al., 2011 ▶). TNF acts as a homeostatic agent, but can cause pathology if produced excessively (Clark et al., 2006 ▶), by stimulating other factors such as NOI and ROI (Richards, 1997 ▶). Besides, early pro-inflammatory cytokine responses may enhance immunity against parasitic infection, while long-term or late onset anti-inflammatory responses are associated with severe and complicated *P. falciparum* malaria (Sinha et al., 2008 ▶; Torre et al., 2009 ▶). 

Also, IFN- is an important macrophage activating factor involved in the immune response to malaria. It is produced by both CD8+ and CD4+ T lymphocytes in specific responses to antigen as well as by NK cells in a nonspecific manner. The regulation of IFN- secretion by these lymphocytes is mostly controlled by the cytokine IL-12. The targets of IFN- during malaria are monocytes/macrophages, neutrophils, Th2 cells (inhibits proliferation) and parasite infected hepatocytes. IFN- activated macrophages are stimulated to release TNF, ROI, NOI, TGF , IL-1 and IL-6. These compounds have shown individually or in combination to kill and inhibit the growth of parasites *in vitro* and in mouse models. The ability of IFN- to stimulate macrophages to release factors such as TNF may be related to its role in cerebral malaria, since antibodies to IFN- have been reported to prevent experimental cerebral malaria in mice (Richards, 1997 ▶).

Nitric oxide (NO) is an endothelium – derived relaxation factor and one of the important anti-parasitic chemicals generated by macrophages during innate immune responses (Awasthi et al., 2003 ▶). NO has been suggested to play a key role in the pathogenesis of cerebral malaria. It can exert beneficial and detrimental effects in malaria depending on the timing and amounts of its production and the biological milieu in which it is released. For instance, at low concentration, NO may act as an anti-inflammatory molecule by eliciting macrophages cytotoxicity against the invading parasites. However, at high concentration, NO may become cytotoxic to both the invading parasites and the host’s own cells (Awasthi et al., 2003 ▶; Sobolewski et al., 2005 ▶). 

Increased NO concentration may exert cytotoxicity through a number of different mechanisms such as inhibition of enzymes with iron-sulfur centers activity through the formation of iron-sulfur–nytrosyl derivatives--reaction with superoxide anions leading to the production of potent oxidizing agents such as peroxynitrite and lipid peroxidation of cell membranes. NO may also deaminate some DNA bases that may result in mutations and strand breaks. Hence, NO requires tight regulation to prevent unwanted side effects (Awasthi et al., 2003 ▶). Increased NO production have been reported during parasitic infections and this is mediated by upregulated expression of the inducible NO synthase (INOS or NOS2) in response to secretion of pro-inflammatory cytokines during infections and/or exposure to specific parasite antigens (Brunet, 2001 ▶).

In this study, the marked increase (p<0.05) in cytokines level in the infected control mice and in the infected mice treated with a lower dose of the extract (100mg/kg) may be attributed to increased schizogony and fever paroxysms resulting in increased TNF-, IFN- and NO levels. The cytokines may affect the receptors expression on vascular endothelium directly by either redistributing or upregulating the receptors; thus, increasing the cytokines level (Heddini, 2002 ▶). On the other hand, the treatment of the infected mice with either higher doses of the extract (300–1000 mg/kg) or CQ (20mg/kg) might have alleviated the inflammatory effect of the *P. berghei* parasite by direct inhibitory effect on cytokines production, including TNF-, IFN-, NO and also might have generated many kinds of antioxidants *in vivo* with protective activities against protozoal infections (Awasthi et al., 2003 ▶). 

The significant reduction in hemoglobin level especially in the untreated infected mice and the extract treated mice (at a lower dose 100mg/kg) in this study, suggests anaemia. Moreso, hematological abnormalities are considered as hallmark of malaria infection due to higher levels of parasitaemia. Several papers have also reported peripheral blood changes, including anaemia, as a common presentation. Anemia may be normochromic and normocytic or microcytic hypochromic in the majority of cases. The pathogenesis of anaemia in malaria still remains unclear. It has been adduced to several factors including hemolysis of parasitized and unparasitized erythrocytes, improper formation of red blood cells as well as anaemia of chronic disease (Clark et al 2006 ▶).

Furthermore, TNF- has been implicated in bone marrow depression leading to ineffective erythropoiesis by inhibiting the growth and differentiation of erythroid progenitor cells. Moreover, TNF-induced dyserythropoiesis has been confirmed in rats and mice expressing high levels of TNF- and cytokines induced by malaria products which are major determinants of hemoglobin deficiency and, thus, the rate at which oxygen reaches mitochondria in malaria (Clark et al 2006 ▶). The mechanism of TNF-induced damage to human bone marrow cells has been suggested to be nitric oxide generated by iNOS induced by TNF- (Clark et al., 2006 ▶; Yeo et al., 2009 ▶). Nonetheless, incidence of anemia in malaria infection is said to correlate with the severity of the infection (Bashawri et al 2002 ▶). 

The significantly higher concentration (p<0.05) of malonaldehyde (MDA), a lipid peroxidation product, in the serum of the non-treated infected group as well as in the lower dose (100mg/kg) treated mice in this study, reflects an increase in peroxidation of their membrane lipids and, thus, the severity of the malaria infection. This may be explained by the fact that *Plasmodium*-infected erythrocytes are under high endogenous oxidative stress and decreased antioxidant enzymes in parasitized erythrocytes (Egwunyenga et al, 2004 ▶). 

Central to the generation of oxidative stress is the degradation of host haemoglobin by the parasite. Haemoglobin represents the major source of amino acids for the parasite, but its degradation in an acidic food vacuole results in the production of redox active by-products, toxic free-haem and reactive oxygen species (ROS), conferring oxidative insult on the host cell. Apart from this metabolically derived oxidative stress, the production of ROS by the host immune system adds to the overall oxidative burden of the parasitized cells (Becker et al, 2004). Several studies have also reported evidence showing some physicochemical changes in the membrane of the erythrocyte induced by oxidative peroxidation and hemolysis seen in malaria (Das and Nanda, 1999 ▶; Egwunyenga et al., 2004 ▶). Golenser and Chevion, (1989) ▶ earlier reported an increase in the concentration of malonaldehyde in the blood of *P. berghei *exposed mice. 

The reduced MDA concentration (p < 0.05) in the infected mice that were treated with higher doses of the extract may be attributed to the protective effect of the leaf extract against membrane damage due to ROS. *V. amygdalina* leaf has been previously reported to possess antioxidant activity due to presence of some phytochemicals such as flavonoids, sesquiterpene lactones and steroidal saponins (Erasto et al., 2007 ▶). The HPTLC chemical finger-print profile of the plant extract further attested to the presence of these phytochemical compounds which was evidenced by several though unidentified absorption peaks. From literature, phenolic compounds (such as flavonoids) have been suggested to be responsible for the antioxidative activities of edible and non-edible plant products by offering protection against harmful ROS (Erasto et al., 2007 ▶; Omoregie and Osagie, 2011 ▶). 

The overall findings from this study revealed the parasite suppressive effect of ethanol extract of *V. amygdalina* leaf in *P. berghei* infected mice in a dose-dependent manner. The result is consistent with previous works on the antimalarial activity of *V. amygdalina* both *in vitro* (Atawa et al., 2003 ▶; Tona et al., 2004 ▶; Challand and Willcox, 2009 ▶; Omoregie et al., 2011 ▶) and *in vivo* (Abosi and Raseroka, 2003 ▶; Challand and Willcox, 2009 ▶). The antimalarial activity of *V. amygdalina* may be attributed to the presence of several sesquiterpene lactones compounds, chemically related to artemisinin: vernolide, vernodalin, hydroxyvernolide and vernodalol. It also contains the steroidal glucosides (vernoniosides B_1_-B_3_ and vernoniosides A_1_-A_4_), which gives the plant its bitter taste (Kraft et al., 2003 ▶; Challand and Willcox, 2009 ▶). Besides, the antimalarial activities of these compounds are well documented in the literature (Kraft et al., 2003 ▶; Tona et al., 2004 ▶; Challand and Willcox, 2009 ▶). 

In addition to the sesquiterpene lactones and steroidal glycosides, other constituents could be present in the leaf extract that may provide therapeutic properties other than antiparasitic effects, including antipyretic and immune-modulatory. Although several scientific papers have reported on the antiplasmodial activity of *V. amygdalina*, this study reports for the first time the antioxidative and immuno-modulatory activities of ethanol extract of *V. amygdalina* leaf in *P. berghei* infected mice. The parasite suppressive effect of the ethanol extract of the plant is consistent with local claims on the efficacy of the plant in the treatment of malaria and may form one of the components of future combination drugs designed to reduce the level of malaria transmission at the community level.
